# 9 Environmental Contamination Reduction Following Standard, Enhanced, and a Novel Cleaning Protocol in a Burn Center

**DOI:** 10.1093/jbcr/irac012.013

**Published:** 2022-03-23

**Authors:** Anjay Khandelwal, Michael Bigham

**Affiliations:** Akron Children's Hospital, Akron, Ohio; Akron Children's Hospital, Akron, Ohio

## Abstract

**Introduction:**

Burn patients are extremely vulnerable to hospital acquired infections, some of which may originate from environmental surfaces. Enhanced cleaning practices of surfaces varies from hospital to hospital. In a burn unit, patients are frequently exposed to multiple environments including the patient room, hydrotherapy and treatment rooms (treatment spaces), and the operating room. We sought to evaluate the efficacy of current protocols, including standard cleaning (SC) and cleaning with ultraviolet light application (C+UV) in the patient rooms and treatment spaces, and compare to a novel Hybrid Hydrogen Peroxide (HHP) fogging system in the patient rooms.

**Methods:**

This study was conducted at a regional 12-bed verified adult and pediatric burn center that functions as an intensive care unit and step-down unit, where patients stay during their entire admission. Data from December 2020 to June 2021 was collected from rooms following 17 patient discharges with a minimum of a 2-week hospital stay. Data was collected from 5 preset locations in the patient rooms following either SC or C+UV and after HHP fogging. Baseline data was also collected from 20 preset locations in the treatment spaces following SC and C+UV. Quantitative and qualitative counts (aerobic colony county [ACC], adenosine triphosphate [ATP]), hydrogen peroxide chemical indicators (CIs) and bacterial spore biological indicators (*G. stearothermophilus* 1x10^6^ spores) were measured.

**Results:**

No statistically significant difference between SC and C+UV was seen (mean ACC 7.16 and 6.35 respectively; p=0.186). ACC levels were reduced following HHP fogging demonstrating a 98% improvement over SC and C+UV (mean ACC 0.14; p< 0.0001). ATP demonstrated an 88% reduction after HHP fog without manual cleaning. Biological indicators confirmed a 1x10^6^ reduction of bacterial spores, and CIs verified a thorough migration of HHP fog throughout the patient room. Baseline sampling of the treatment spaces following SC and C+UV resulted in a range of 0-70 ACC over 38 swabbed locations, consistent with ACC levels measured in patient rooms (range 0-153). An industry-known location for high bioburden, one sink backsplash swabbed on two separate occasions resulted in >5,700 ACC.

**Conclusions:**

In patient rooms, HHP fogging resulted in a significant reduction in ACC compared to current protocols of SC and C+UV. Baseline sampling of the treatment spaces resulted in ACC levels similar or greater in range to those seen in the patient room, indicating a similar reduction in bioburden levels could be achieved through implementation of HHP fogging. The efficacy and feasibility of HHP fogging in a patient room setting within a burn unit suggests that a protocol for the use of HHP fogging for treatment spaces will likewise be beneficial.

